# Genetic association study of fatal pulmonary embolism

**DOI:** 10.1007/s00414-020-02441-7

**Published:** 2020-10-30

**Authors:** Lisa Meißner, Peter Schürmann, Thilo Dörk, Lars Hagemeier, Michael Klintschar

**Affiliations:** 1 Institute of Legal Medicine, Hannover Medical School, Carl-Neuberg-Str.1, 30625 Hannover, Germany; 2 Gynaecology Research Unit, Department of Obstetrics and Gynaecology, Hannover Medical School, Carl-Neuberg-Str.1, 30625 Hannover, Germany

**Keywords:** Pulmonary embolism, Venous thromboembolism, Deep vein thrombosis, Sudden death

## Abstract

Pulmonary embolism (PE) is a complex multi-factorial disease and represents one manifestation of venous thromboembolism (VTE). Most commonly PE constitutes a complication of VTE’s other clinical presentation deep vein thrombosis (DVT). The majority of studies concerning risk factors do not distinguish between PE and DVT. The risk factors are often estimated to be alike, but the prevalence and the risk associated with the major genetic factor Factor V Leiden differ between the two disease states. We have investigated the association of 22 SNPs with PE in 185 PE case and 375 healthy control subjects. At *p* = 0.05, eight SNPs presented with nominally significant evidence of association (EOA), although no significantly different genotype distributions remained between cases and controls after Bonferroni correction. Three of these variants (rs1800790, rs3813948, rs6025) showed EOA in the main analysis, and five variants (rs169713, rs1801131, rs4524, rs5985 and rs8176592) demonstrated EOAs in subgroups. Genomic variation modulating Factor V, Factor XIII, Beta fibrinogen (FGB), TFPI or HIVEP1 should be worth to be followed in subsequent studies. The findings of this study support the view that PE represents a complex disease with many factors contributing relatively small effects. Larger sample sizes will be required to reliably detect these small effects.

**Electronic supplementary material:**

The online version of this article (10.1007/s00414-020-02441-7) contains supplementary material, which is available to authorized users.

## Introduction

Pulmonary embolism (PE) is one manifestation of venous thromboembolism (VTE) and a potentially lethal complication of VTE’s other clinical presentation deep vein thrombosis (DVT). Approximately 65% of VTE patients display only DVT, whereas around 30% of VTE patients also manifest PE [1].

PE diagnosis is challenging due to its variable and often non-specific clinical presentation. Silent PE develops in around 50% of patients with DVT and autopsy reports demonstrated that only 30–45% of all PE cases were diagnosed prior to death. The annual incidence of PE is estimated to be about 50–100 per 100,000 individuals and increases with age [2, 3]. Males are more often affected than females. However, women above 75 years display an incidence of more than 500 cases per 100,000 individuals [4]. In forensic practice, pulmonary embolism is relatively common, either as sudden unexpected death or in the context of potential maltreatment. In our own praxis, we observe pulmonary embolism in ca. 4% of all medicolegal autopsies.

Pulmonary embolism is a complex multi-factorial disease which is caused by external as well as multiple genetic factors. However, most studies on risk factors were conducted analysing associations with VTE and not PE in particular [5]. External risk factors can be divided into permanent, patient-associated risk factors and, temporary circumstances like immobilisation [2]. In 20–50% of the cases, PE occurs in absence of these risk factors [6, 7]. Moreover, twin and family studies suggested a major genetic constituent risk to be related to VTE by demonstrating an increased risk for individuals with affected siblings and a strong heritability [8–10]. The genetic predisposition to elevated risk of VTE is described by the term thrombophilia, which is often associated with gene variations of the physiological coagulation cascade [11]. Minor changes in the balance between the system of coagulation and fibrinolysis might cause thrombus formation. These changes can occur through mutations in genes coding for coagulation factors and inhibitors, which lead to altered levels or functional losses of associated proteins [12].

Until the beginning of 2000, only few gene loci were robustly identified to be associated with VTE. Most of these variations represent strong genetic risk factors increasing the risk of VTE in heterozygous carriers almost 10-fold, such as deficiencies of antithrombin or protein C [5, 13]. However, these genotypes occur in less than 1% of the population and thus represent only few patients suffering from VTE [14]. Subsequently, new loci, presenting weaker risk factors but higher frequencies, were identified through studies of candidate genes [15] and genome-wide association studies (GWAS) [14, 16–18].

Since PE is usually regarded as a complication of DVT, their inherited risk factors are often regarded to be similar, but the prevalence and the risk associated with some risk factors such as Factor V Leiden or activated protein C resistance differ between DVT and PE [19, 20]. The Factor V Leiden paradox states that the rs6025 polymorphism increases the risk of DVT much more than the risk of PE [20]. These findings emphasize the necessity of considering DVT and PE as different phenotypes in VTE genetic studies. Only one study investigating a broad panel of genetic VTE risk factors in patients with fatal PE has been reported so far [21]. Brandimarti et al. conducted a study in an Italian population investigating associations between 12 thrombophilic risk factors and VTE in 44 individuals who died from PE. Strong associations between PE and the polymorphisms C677T (MTHFR), Pai-1 4G/5G and ITGB3 were found [21].

The present study aims to replicate the results of the aforementioned study using a considerably larger sample size. Furthermore, this study intends to evaluate the association of additional SNPs which were robustly associated with VTE or DVT in previous studies in subjects who died from PE.

## Material and methods

### Investigated subjects

Patients who died from PE were identified by a review of the autopsy reports of the Department of Forensic Medicine at Hannover Medical School and its remote station in Oldenburg. The case group comprised samples from 185 Caucasian adults (mean age 62 years) for whom PE was diagnosed post mortem. The group comprises samples from 90 male and 92 female subjects and three individuals without documented gender. For four samples, no information was given about the age. Concerning VTE risk factors, 90% of all case samples presented at least one external risk factor. Of all samples, 51% exhibit at least one of the risk factors surgery, tumour or obesity. Blood samples on filter paper or cotton swabs were obtained during autopsy of the PE cases and stored with the respective file at room temperature.

The control group was composed of 375 samples from healthy Caucasian individuals (mean age 36 years) which had been voluntary blood donors at the Transfusion Department of Hannover Medical School. DNA extracts were obtained from the Hannover Breast Cancer Study and Hannover Prostate Cancer Study of the Gynaecology Research Unit (Hannover Medical School). The group comprised samples from 189 female and 186 male blood donors.

### DNA extraction

DNA was extracted using the QIAmp®DNA Mini Kit (QIAgen, Hilden, Germany). In order to achieve from the filter paper the recommended DNA concentration of 10 ng/ml for SNPtype genotyping on the Fluidigm Dynamic Array integrated fluidic circuits (IFCs), pilot experiments using different amounts of elution buffer were conducted. Results were compared utilizing Thermo Scientific NanoDrop 8000. Best reproducible DNA yields were reached eluting with 50 μl and three times repeated elutions.

### Variant selection

A total of 27 candidate gene variants were selected based on previous literature. Twelve SNPs used in the study conducted by Brandimarti et al. [21] were chosen in order to examine whether their results can be replicated in our larger sample. The National Center for Biotechnology Information (NCBI) PubMed database was searched for papers assessing other relevant VTE and PE associated SNPs or SNPs affecting the coagulation of blood and their prevalence in the general population as well as in VTE-exposed populations. Used search terms were genetics, risk factors, thrombophilia, venous thrombosis, pulmonary embolism and the polymorphisms itselves (date of research: May‑July 2018). Due to power calculations, only SNPs exhibiting a minor allele frequency (MAF) higher than 1% were chosen. For 26 of 27 initially chosen SNPs, SNPtype genotyping assays using the Fluidigm® approach could be successfully designed. These SNPs were tested for proper clustering utilizing samples from healthy controls for genetic studies of the Gynaecology Research Unit (Hannover Medical School). SNPs displaying poor clustering in the wet lab testing were excluded from further analyses. Altogether 22 SNPs, including nine of those SNPs tested by Brandimarti et al. [21], could be successfully genotyped. Table 1 displays the SNPs used in this study.

**Table 1:** Single nucleotide polymorphisms associated with VTE or hereditary thrombophilia

Gene	refSNP accession no.	Effect allele	Reference publication
*MTHFR*	rs1801133	T	Brandimarti et al. [21]
*MTHFR*	rs1801131	C	Brandimarti et al. [21]
*F5*	rs6025	A	Brandimarti et al. [21]
*F2*	rs1799963	A	Brandimarti et al. [21]
*FGB*	rs1800790	A	Brandimarti et al. [21]
*F13A*	rs5985	A	Brandimarti et al. [21]
*ITGB3*	rs5918	C	Brandimarti et al. [21]
*TFPI*	rs8176592	C	Brandimarti et al. [21]
*TFPI*	rs10931292	C	Brandimarti et al. [21]
*F5*	rs4524	G	Germain et al. [22]
*FGG*	rs2066865	T	Germain et al. [16]
*FGG*	rs6536024	C	Tang et al. [18]
*ABO*	rs8176719	Deletion	Heit et al. [17]
*ABO*	rs2519093(v2)	A	Heit et al. [17]
*ABO*	rs529565(v2)	C	Germain et al. [22]
*F11*	rs4253399	G	Tang et al. [18]
*F11*	rs4253417	C	Germain et al. [22]; Sennblad et al. [23]
*F11*	Rs2036914	C	Austin et al. [24]; De Haan et al. [25]
*SLC44A2*	rs2288904	T	Germain et al. [22]
*HIVEP1*	rs169713	C	Morange et al. [26]
*C4BPB*	rs3813948	G	Buil et al. [27]
*TC2N*	rs1884841	T	Morange et al. [28]

### Genotyping

Amplification and genotyping were performed using allele-specific SNPtype assays on 192.24 Dynamic Array IFCs using the BioMark EP1 Real-Time PCR System (Fluidigm Corp., South San Francisco, CA, USA) as previously described [29]. In short, assays composed of FAM or HEX fluorescence-labelled primer, detecting respectively one allele of each SNP, and an untagged locus-specific reverse primer were designed by Fluidigm for each SNP [30] (online supplementary material: Table 1).

Specific target amplification (STA) as a preamplification step was performed for all case and control samples as well as two non-template controls per array in a 96-well thermocycler using 10× SNPtype STA Primer Pool, 2× Multiplex PCR Master Mix (Qiagen, Hilden, Germany) and genomic DNA. Thermocycling conditions were 15 s at 95 °C followed by 14 cycles of 15 s at 95 °C and 4 min at 60 °C. The STA products were then diluted 1:100 in DNA suspension buffer, a low-EDTA TE buffer (10 mM Tris, 0.1 mM EDTA; pH 8.0), and stored at − 20 °C.

The Sample Pre-Mix per 192 samples was prepared by combining 540 μl Biotium 2x Fast Probe Master Mix, 54 μl 20x SNPtype Sample Loading Reagent, 18 μl 60x SNPtype Reagent, 7 μl ROX and 12 μl PCR-certified water. After vortexing and centrifugation, the sample mix for each sample was prepared by adding 2.6 μl Sample Pre-Mix to 2 μl of the diluted STA product. For each SNP, the 10x Assay mix was prepared by 2 μl 2× Assay Loading Reagent (Fluidigm), 1.2 μl PCR-certified water, and 1 μl SNPtype assay mix (Fluidigm).

The Dynamic Array IFCs were each loaded with 190 times 3 μl prepared sample pre-mix, two NTCs and the 10x assays. Loading of the array IFC as well as mixing and thermocycling followed the manufacturer’s instructions. Mixing of the samples and assays was conducted with the IFC Controller RX (Fluidigm). Thermocycling was performed on the BioMark EP1 Real-Time PCR System and run according to the default protocol.

In total, five Dynamic Array IFCs were run. For quality control, 16.5% of all samples were run in duplicates.

### Data analysis

Resulting data from the array runs was displayed, automatically analysed and manually checked using the data analysis software Fluidigm SNP Genotyping Analysis [30]. Samples with more than 4 “no calls” or less than 90% concordance with their duplicates were eliminated from the study (*n* = 26). SNPtype assays were checked for call rates below 90% or poor clustering (none excluded). Moreover, Hardy-Weinberg equilibrium (HWE) was examined using the *χ*2 tests. Assays displaying significant deviations were also omitted (*n* = 2) (rs2227589, rs710446).

Statistical analyses were conducted using logistic regression with STATA® 12.0 on distinct results for 22 SNPs, overall and within subgroups. Genotype frequencies from cases and controls were compared and tested for significant differences using a univariate linear regression analysis with case-control status as the outcome variable under an additive risk model. In addition, a stratified analysis according to known environmental risk factors of the case samples was conducted. For this purpose, samples were stratified in eight different subcategories in order to filter out those case samples of patients whose death is more likely related to environmental risk factors than genetic risk factors and thus increase the proportion of case samples that died because of a stronger genetic influence. These categories included age over 50, sex, body-mass index (BMI) above 30, recent surgery and tumours. We tested all cases without known risk factors against all controls, as well as all cases without tumour, operation and BMI below 30 against all controls. Results were expressed as odds ratios (OR) with 95% confidence intervals (CI) and two-sided *p* values. In case of zero fields for a particular SNP, we tested for significant differences using the Fisher’s exact test.

In order to avoid increased false positivity rates due to multiple comparisons [31], this study also utilized the Bonferroni correction for all *p* values. The significance level after multiple testing was considered 0.002 [32].

Mantel-Haenszel pooled OR after a fixed-effect meta-analysis was conducted for the combined data set of Brandimarti et al. [21] and our study results using the metan command in STAT12.0 with an increment of 0.1 to account for zero fields. As the meta-analysis comprised nine different variants, the level of statistical significance was adjusted accordingly (*α* = 0.006).

## Results

Genotyping achieved call rates between 95.7% (rs8176592) and 99.1% (rs3813948) as well as an overall call rate of 98.1% for the 560 samples that were genotyped for 22 SNPs.

At a significance level of 0.05, eight of 22 SNPs presented nominally significant results in either the main or the eight subgroup analyses before the correction for multiple testing. After utilizing the multiple-comparison Bonferroni correction for the main analysis (*p* = 0.0017), no significantly different genotype distributions remained between cases and controls. However, *p* ≤ 0.05 was regarded in this study as an evidence of association (EOA) suggesting noteworthiness for the investigation of causes for PE. The results for those variants demonstrating *p* < 0.05 in the logistic regression analyses are depicted in Table 2. Three of 22 SNPs demonstrated EOAs in the main analysis (rs1800790, rs3813948, rs6025). The five additional polymorphisms rs169713, rs1801131, rs4524, rs5985 and rs8176592 presented EOA in subgroup analyses. Values ranged between *p* = 0.006 (rs169713 in “all cases without surgery, tumour or BMI below 30 kg/m^2^ vs. all controls”; rs6025 in “all cases without tumour vs. all controls”) and *p* = 0.05 (rs1801131 in “cases without known risk factors vs. all controls”). EOAs were found for rs169713 in two subgroups, for rs1800790 in four, for rs1801131 in one, for rs3813948 in four, for rs4524 in one, for rs8176592 in one and for rs6025 in five subgroups (Table 2).When being compared to literature [33], the MAF of our control group presented deviations ranging from 0.043 (rs1801133) to 11.2% (rs169713) (online supplementary material: Table 1).

**Table 2:** Selected results from the logistic regression for the main- and subgroup analyses according to risk factors

Analyse	Gene	Marker	OR	*p* value	95% confidence interval	
Main analysis						
All cases vs. all controls						
*FGB*		rs1800790	0.7078	0.028	0.52	0.96
*C4BPB*		rs3813948	0.4592	0.016	0.24	0.87
*F5*		rs6025	2.1312	0.010	1.2	3.79
Subgroup analyses						
Male cases vs. all controls						
*HIVEP1*		rs169713	1.5650	0.042	1.02	2.41
*F5*		rs4524	0.6323	0.035	0.41	0.97
Female cases vs. all controls						
*FGB*		rs1800790	0.5906	0.027	0.37	0.94
*C4BPB*		rs3813948	0.3305	0.028	0.12	0.89
*F5*		rs6025	2.7021	0.013	1.23	5.93
Cases with PE at age < 50 years vs. all controls						
*F13A*		rs5985	0.4676	0.009	0.27	0.83
*FGG*		rs6536024	0.5552	0.013	0.35	0.88
Cases with BMI > 30 vs. all controls						
*TFPI*		rs10931292	0.4020	0.019	0.19	0.86
Cases without known risk factors vs. all controls						
*MTHFR*		rs1801131	0.3456	0.050	0.12	1.00
*TFPI*		rs8176592	0.3298	0.039	0.12	0.95
All cases without surgery vs. all controls						
*C4BPB*		rs3813948	0.3906	0.012	0.19	0.81
*F5*		rs6025	2.2277	0.009	1.22	4.07
All cases without tumour vs. all controls						
*FGB*		rs1800790	0.7081	0.035	0.51	0.98
*C4BPB*		rs3813948	0.4704	0.024	0.24	0.91
*F5*		rs6025	2.2821	0.006	1.27	4.09
All cases without operation, tumour and with BMI below 30 kg/m^2^ vs. all controls						
*HIVEP1*		rs169713	1.7373	0.006	1.18	2.57
*FGB*		rs1800790	0.6049	0.022	0.39	0.92
*TC2N*		rs1884841	0.6326	0.012	0.44	0.91
*F5*		rs6025	2.6133	0.007	1.3	5.26

Bonferroni correction was used to correct for multiple comparisons. After correction, no SNPs remained nominally significant. *α* = 0.05 was used as an evidence of association. For rs1799963, no results were obtained in the subgroups “cases with PE before 50 years vs. all controls” and “cases without known risk factors vs. all controls” because none of the residual case samples showed deviations from the wild-type allele and the odds ratios could not be calculated with zero fields

A meta-analysis of our results with previously published results from Brandimarti et al. [21] showed for some previously proposed SNPs opposing or strongly deviating effects, with significant heterogeneity between studies for rs8176592 (*p*_het_ 0.028) and rs5918 (*p*_het_ 0.001). For rs1801133 and rs10931292, both studies showed similar tendencies though the meta-analysis did not reach statistical significance (Table 3). From our own study, *FGB* (rs1800790, *p*_meta_ = 0.068) and *F5* (rs6025, *p*_met_ = 0.064) remained with marginal evidence of association.

**Table 3:** Meta-analysis with data from Brandimarti et al. [21]

Variant ID	Brandimarti et al. [21]	This study	Meta OR (95% CI)	*p*_meta_	*p*_het_
rs1801133	0.879 (0.522; 1.479)	0.910 (0.698; 1.186)	0.903 (0.713; 1.144)	0.398	0.907
rs1801131	0.517 (0.294; 0.911)	0.993 (0.756; 1.304)	0.872 (0.684; 1.113)	0.273	0.042
rs6025	0.032 (0.000; 16.266)	2.114 (1.196; 3.734)	1.660 (0.971; 2.836)	0.064	0.134
rs1799963	0.032 (0.000; 16.266)	0.867 (0.330; 2.274)	0.600 (0.241; 1.490)	0.271	0.234
rs1800790	2.398 (0.676; 8.501)	0.712 (0.524; 0.966)	0.758 (0.564; 1.018)	0.068	0.068
rs5985	1.988 (0.590; 6.694)	1.042 (0.793; 1.369)	1.073 (0.822; 1.401)	0.603	0.309
rs5918	9.571 (2.600; 35.238)	0.897 (0.626; 1.285)	1.107 (0.794; 1.545)	0.548	0.001
rs8176592	1.701 (1.013; 2.857)	0.883 (0.673; 1.159)	1.014 (0.798; 1.289)	0.907	0.028
rs10931292	0.619 (0.257; 1.489)	0.791 (0.534; 1.170)	0.758 (0.530; 1.083)	0.128	0.617

## Discussion and conclusion

Clinical medicine as well as forensic post mortem investigations would benefit from well-defined genetic risk factors for PE. However, almost all studies on the association of gene loci with VTE up to now do not differentiate between PE and DVT [24, 27, 34]. There is however a big difference in consequences for a patient between DVT and the potentially deleterious complication of PE. Furthermore, it is well known that, fortunately, only a minority of patients with increased DVT risk, e.g. from Factor V Leiden, succumb from PE, a phenomenon described as Factor V Leiden paradox. On the other hand, studies on risk factors for PE are mostly focused on external risk factors and not on candidate genes from studies on VTE, which results in an insufficient knowledge concerning their influence on PE (e.g. [35]).

The only study that focuses on genetic variants in deaths from PE we are aware of is that by Brandimarti et al. [21] that found significant differences between a group of persons deceased from PE and a control group for SNPs in MTHFR*C6777T (rs1801133), SERPINC1C*4G/5G (rs397832688) and ITGB3*T196C (rs5918). However, the groups tested in this study were small: 44 persons who died from PE and 102 controls. While rs397832688 was not amenable to our SNPtype assay design, our study of 375 controls and 185 cases could not reproduce a significant difference in genotype frequencies for the two SNPs MTHFR*C6777T (rs1801133) and ITGB3*T196C (rs5918), respectively, between PE cases and controls reported by Brandimarti et al. [21]. These differences in results may be explained by the small number of PE cases and controls in the initial study [21]. Small sample sizes may lead to overestimations of the particular effect size [36].The overestimation of the effect size can, in turn, result in overestimations of the power of replication studies, which might have affected the a priori calculated power for a few variants of the present study [37]. For instance, the statistical power for rs5918 in our study design was determined a priori on the basis of the genotyping results of Brandimarti et al. [21] as being close to 100% to detect a significant difference at *p* < 0.05 with the given risk estimate and study size. However, the statistical power for the rs5918 polymorphism based on the truly obtained genotype frequencies amounts to 16%. The post hoc calculated power for the other SNPs tested in our study ranges between 1 (rs1801133) and 62% (rs1800790). This emphasizes that, according to Ziegler et al. [38], extensive sample sizes are necessary for a reliable detection of the small effects which are expected for the contributing factors in complex diseases like PE. Most of the variants that we analysed showed only small effects (see OR and their 95% CI in Table 2). In comparison to Brandimarti et al. [21], we used approximately the 4-fold number of cases as well as controls, but for a reliable determination and replication of the small effect sizes, the study size still would need to be strongly increased.

In our study, rs1800790, rs3813948 and rs6025 demonstrated EOA for the main logistic regression analysis. The Factor V Leiden mutation (rs6025) displayed similar effects in the subgroup analyses indicating a rather small effect of stratification. According to the FV Leiden paradox, the risk of DVT is substantially increased in carriers of the *FV* polymorphisms, whereas the risk for PE is only mildly increased [39]. The rs6025 was not found in any of the 44 cases studied by Brandimarti et al.; however, Van Stralen et al. [39] demonstrated in carriers of the Factor V Leiden mutation an approximately 8-fold increased risk of DVT (OR 7.7, 95% CI 3.9–15.3), whereas the risk of PE was small (OR 1.4, 95% CI 0.7‑2.7). Our study is consistent with an about 2-fold increased risk of PE in carriers of the mutation which supports a role of this mutation for PE but is well in line with the results from the aforementioned study.

The *FGB* polymorphism 455 G/A (rs1800790) has been associated with higher levels of fibrinogen, which in turn suggests an association of the variant with a hypercoagulable state as well as thrombosis [40]. Studies could not agree whether the polymorphism exerts a protecting role or none at all. Whereas some studies [40, 41] detected a lower VTE risk to be associated with the polymorphism, another [42] found no association between the mutation and PE. Brandimarti et al. [21] could not demonstrate significant differences, but our study demonstrated an EOA for the polymorphism in the main analysis and three subgroup analyses (see Table 2). The computed ORs indicate a protective effect of the rare allele.

The protein C4BPB binds to protein S and thus presents a main element of the coagulation and fibrinolysis cascade. A previous study demonstrated a slightly increased risk associated with the polymorphism rs3813948 located in the *C4BPB* gene [27]. However, the results from our study indicate the opposite effect, demonstrating a rather protective effect of the polymorphism. A possible explanation for the opposing results is that Buil et al. [27] analysed the association with VTE without differentiation in DVT and PE cases, whereas our study examined those polymorphisms only in PE deaths. In this case, the results would suggest strongly differing risks associated with the polymorphism for PE and DVT, which need to be examined in further studies.

The polymorphisms at rs169713, rs1801131, rs4524, rs5985 and rs8176592 presented EOA in the subgroup analyses that we performed in order to maximize the effect of the genetic variant by enriching the susceptible group through exclusion of externally influenced cases, as proposed [38].

Franco et al. [43] demonstrated a protective role of the rare SNP allele for the Factor XIII p.Val34Leu polymorphism (rs5985). The subjects who did not harbour the rare allele presented a 6-fold increased risk of developing DVT. Our study results suggest the rare allele of this polymorphism to exert a protective role not only for DVT but possibly also for PE.

Lincz et al. [44] examined the activity of the TFPI and frequencies of *TFPI* polymorphisms in patients presenting Factor V Leiden and a manifestation of VTE (rs8176592). They found a strongly increased VTE risk in carriers of the polymorphism in the presence of Factor V Leiden. However, most other studies demonstrated a small protective effect of the polymorphism on the VTE risk by increasing the TFPI plasma level [32]. In the present study, we replicated the protective effect of the T33C mutation. Individuals without environmental risk factors, which do not exhibit the cysteine variant, displayed an about 3-fold increased risk of dying of PE.

As the first VTE susceptibility locus outside the traditional coagulation cascade, the rs169713 polymorphism of the *HIVEP1* locus was significantly associated with VTE risk in a study by Morange et al. [26]. HIVEP1 affects the transcriptional regulation of target genes associated with inflammatory processes [5]. By increasing the damage on endothelial cells and enhancing hypercoagulability, inflammation is considered to act as a contributor to the VTE risk [45]. The results of our study support the aforementioned result, by demonstrating notable differences in two subgroup analyses. Moreover, our results suggest that the rare allele is associated with a slightly higher risk of PE than VTE in general.

Associations of the A1298C (rs1801131) and C677T (rs1801133) variants of the *MTHFR* gene and an increased VTE risk were found by Liu et al. [46]. Brandimarti et al. [21] examined these two polymorphisms but their results did not demonstrate significant differences of the polymorphisms in cases and controls. Furthermore, a study on a Chinese population [47] demonstrated significant results for the C677T variant of the *MTHFR* gene and VTE. Our study demonstrated evidence for differences in genotype frequencies of cases and controls for the A1298C variant within the subgroup analysis of cases without known risk factors. The C-allele yielded a protective effect on the PE risk. Our results suggest that subjects without known risk factors that do not exhibit the C-allele may display an almost 3-fold increased risk of developing PE.

The rs4524 polymorphism of the Factor V gene was associated in a meta-analysis of more than 65.000 individuals with a slightly increased risk for VTE [22]. Moreover, the effect allele was repeatedly associated with an elevated risk of DVT in three Dutch studies conducted by Bezemer et al. [48]. Our results suggest a rather decreased risk, if any, of PE for carriers of the effect allele in male individuals. These results might be in line with the aforementioned Factor V Leiden paradox. Nevertheless, the paradox was only observed as well as analysed for the rs6025 polymorphism [39] and needs to be investigated for the rs4524 mutation.

The results of our study, the largest in terms of sample size ever conducted solely on deaths from PE, may indicate that the risk of death from PE is—at least in part—determined by a number of gene variants. As the evaluation of the impact of these variants is far from conclusively resolved, further analyses in even larger samples would be rewarding. We followed Ziegler et al. [38] who suggested that trying to simplify the respective disease might improve the outcome of association studies. The authors suggest reducing the diseases’ complexity by choosing subgroups of case individuals which are more homogenous. We already demonstrated that for some SNPs, EOAs were only achieved in subgroup analyses. Further increase of the case group would also lead to increased subgroups and thus more distinct results. Moreover, it would be useful to select preferably non-anonymous age-matched controls, who did not develop the disease despite strong external exposure to risk factors.

In summary, our relatively large case-control genetic association study provided supportive evidence for genetic differences at eight candidate risk loci between cases with death from PE and controls, whereas it has refuted some others. Genomic variation modulating Factor V, Factor XIII, FGB, TFPI or HIVEP1 should be worth to be followed in subsequent studies. The results demonstrate evidence for an association; however, more investigations are necessary to prove a causal relationship. Therefore, sample sizes could further be increased and the number of independent tests could be minimized by focusing on the genetic variants demonstrating EOAs. From the forensic point of view, as already mentioned by Brandimarti et al. [21], genetic testing might be especially discriminatory in cases of medical malpractice.

## Electronic supplementary material

ESM 1 (DOCX 22 kb)

ESM 2 (DOCX 18 kb)
