# Supplementary material for: Genetic association study of fatal pulmonary embolism
Source: Int J Legal Med. 2020 Oct 30;135(1):143–51. doi: 10.1007/s00414-020-02441-7 (PMC7782449; doi:10.1007/s00414-020-02441-7)
Supplement: Supplementary file 1 — (DOCX 22 kb) [file 414_2020_2441_MOESM1_ESM.docx]

# Online supplementary material

Table S1: Fluidigm primer information (5' to 3').

| **SNP** | **Allele** | **ASP1** | **ASP2** | **SNP_SEQ** | **ASP1_SEQ** | **ASP2_SEQ** | **LSP_SEQ** | **STA_SEQ** |  |
| --- | --- | --- | --- | --- | --- | --- | --- | --- | --- |
| **rs10931292** | AG | A | G | CTTTCCAGGATTGTTTTTGTAATCAGTTGAAAAAGAATGCTTTTTTCCCCCTAGAGTTAGGGTTTAAAAAATTACATATTTATTATTACACTGTTAATAA[A/G]CTTTTTTGCAGTTTGTTTCTTCTTCAAGTTATTTCATTTGTTTCACCCACTGTCAATGTATTTATTTTTTTTAAAAGTCTGAGGGCTACCGTTGGAGGTC | CTTGAAGAAGAAACAAACTGCAAAAAAGT | TTGAAGAAGAAACAAACTGCAAAAAAGC | GCTTTTTTCCCCCTAGAGTTAGGGT | TGACAGTGGGTGAAACAAATGAAAT | |
| **rs169713** | CT | C | T | GTGTCTGTGACATAGTGAACAGTCGTCTAGGATGACTAGTCAATGCATCTAGTCAATGCTAGCAGCTACTATCATTATTATTCGCACTCTACACAGCCAG[C/T]GTGGGGATTAATGGCATGATGTGCTTTGAGAGGGAAATTTCTCACTAAGCCTATTTTTTGTTTATTTGTTTGTTTAAGAGACAAGATCTCCCTCTGGAAC | CGCACTCTACACAGCCAGC | CGCACTCTACACAGCCAGT | GCACATCATGCCATTAATCCCCA | TCTAGTCAATGCTAGCAGCTACTATC |  |
| **rs1799963** | AG | A | G | AGGGGGCCACTCATATTCTGGGCTCCTGGAACCAATCCCGTGAAAGAATTATTTTTGTGTTTCTAAAACTATGGTTCCCAATAAAAGTGACTCTCAGC[A/G]AGCCTCAATGCTCCCAGTGCTATTCATGGGCAGCTCTCTGGGCTCAGGAAGAGCCAGTAATACTACTGGATAAAGAAGACTTAAGAATCCACCACCTGGT | TCCCAATAAAAGTGACTCTCAGCA | TCCCAATAAAAGTGACTCTCAGCG | GCTGCCCATGAATAGCACTGG | TTGTGTTTCTAAAACTATGGTTCCCA |  |
| **rs1800790** | AG | A | G | TTTCATAGAATAGGGTATGAATTTGTTATTTTGTTATTTTGATTAATGTCTAAAACAAAAGATAAACACATTATGATATAACATTACTATTGATTTTAAT[A/G]GCCCCTTTTGAAATAGAATTATGTCATTGTCAGAAAACATAAGCATTTATGGTATATCATTAATGAGTCACGATTTTAGTGGTTGCCTTGTGAGTAGGTC | CAATGACATAATTCTATTTCAAAAGGGGCT | ATGACATAATTCTATTTCAAAAGGGGCC | ACAAAAGATAAACACATTATGATATAACATTACTATTGAT | CCATAAATGCTTATGTTTTCTGACAATGA |  |
| **rs1801131** | AC | A | C | CCTCTTCTACCTGAAGAGCAAGTCCCCCAAGGAGGAGCTGCTGAAGATGTGGGGGGAGGAGCTGACCAGTGAAG[A/C]AMGTGTCTTTGMAGTCTTYGTTCTTTACCTCTCGGGAGAACCAAACCGGAATGGTCACAAAGTGAGTGATGCTGGAGTGGGGACCCTGGTTCATCCCCTG | GAGGAGCTGACCAGTGAAGA | AGGAGCTGACCAGTGAAGC | TCCGGTTTGGTTCTCCCGAG | CCAAGGAGGAGCTGCTGA |  |
| **rs1801133** | CT | C | T | TGACTGTCATCCCTATTGGCAGGTTACCCCAAAGGCCACCCCGAAGCAGGGAGCTTTGAGGCTGACCTGAAGCACTTGAAGGAGAAGGTGTCTGCGGGAG[C/T]CGATTTCATCATCACGCAGCTTTTCTTTGAGGCTGACACATTCTTCCGCTTTGTGAAGGCATGCACCGACATGGGCATCACTTGCCCCATCGTCCCCGGG | GCTGCGTGATGATGAAATCGG | AGCTGCGTGATGATGAAATCGA | AGGCTGACCTGAAGCACTTGA | TGTGTCAGCCTCAAAGAAAAGC |  |
| **rs1884841** | CT | C | T | ATCATGTGGCTGAACCCAAAATCAACGGGTAGGGAAATATACTCCACTCCTTCAGTGGAAGGAACTTCAAAGTCGTAGCAAAGGGCATGGATACAGTGCA[C/T]GATGAAGTCCTGGGGCCAATACTGTAACCTACTAAAAAGAAGGAATATGACTAGGACTGTGGCTCCAGTAAGTGGAACCACATAGCTTATCTGCAACTGT | AGGGCATGGATACAGTGCAC | AGGGCATGGATACAGTGCAT | GGTTACAGTATTGGCCCCAGGA | AAGGAACTTCAAAGTCGTAGCAAA |  |
| **rs2036914** | CT | C | T | GTTTGGATAAAGAGACGCAATTAGGAAAGGAAAAAGCAGAAGGCTCGTTCCAGACCTGGATGAGATCCTAAAAAGCAGCAGCTTTTGCCAGTAAAGA[C/T]CCTTGAAATGATTCAATTACCCTCAAAGCACTCCTTGTCTCCAAGACAATCACTCATAAGCACAATTCCATTGAAGCCAACGTACCATTTTGTGATTTTC | TTTGAGGGTAATTGAATCATTTCAAGGG | CTTTGAGGGTAATTGAATCATTTCAAGGA | AAGCAGCAGCTTTTGCCAGT | TCTTGGAGACAAGGAGTGCT |  |
| **rs2066865** | CT | C | T | AGACCCCATGTTGAAAACTCCATAACAGTTTTATGCTGATGATAATTTATCTACATGCATTTCAATAAACCTTTTGTTTCCTAAGACTAGATACATGGTA[C/T]CTTTATTGACCATTAAAAACCACCACTTTTTGCCAATTTACCAATTACAATTGGGCAACCATCAGTAGTAATTGAGTCCTCATTTTATGCTAAATGTTAT | CCTTTTGTTTCCTAAGACTAGATACATGGTAC | CCTTTTGTTTCCTAAGACTAGATACATGGTAT | GCAAAAAGTGGTGGTTTTTAATGGTCAA | CATGCATTTCAATAAACCTTTTGTTTCC |  |
| **rs2227589** | AG | A | G | TTTTGTGACCTCCAAAGGACTCACAGGAATGACCTCCAACACCTTTGAGAAGACCAGGCCCTCTCCCTGGTAGTTACAGTCAAAGACCTGTTTGGAAGAC[A/G]TCATTTCAAGTGCTCTCCCTCCCACCCCACCTCTTGGGGTAAGGCCTTTCCTAAGCTACCCCTTGGGTCCCTAGCCTAAGAAACAAGGGGGATGTCATCC | AGTCAAAGACCTGTTTGGAAGACA | GTCAAAGACCTGTTTGGAAGACG | AGGCCTTACCCCAAGAGGTG | CCCTCTCCCTGGTAGTTACA |  |
| **rs2288904** | CT | C | T | CTGAGCCTCTGCAGAGCCTCCCCGTCACCACCTGAGCCTCCTGGGATCCCCTGACTCACAGGGTTTGCTGGGGATGAGGACAGCAGGGCAGTCACCATCT[C/T]GAAGCACCTCAGCCACTCCCTGCCAAGAGAGATGGAGGGAGGTGAGTCCCCAAGTCTAGTACACTGCCACTGCCCATACCTGTGCTCTCAGGACAGTCTA | GCAGGGCAGTCACCATCTC | GCAGGGCAGTCACCATCTT | CTCCATCTCTCTTGGCAGGGA | GACTCACAGGGTTTGCTGG |  |
| **rs2519093 (v2)** | CT | C | T | TACCAGTTCTCCATGATGTGAATTTTCCATTGTATGACTGAACCACAATATCTCAGGGACCCCATAAATATATACACCTACTATGAACCCATAAAATGAAAAACAAAATACTTCTTAGAAAACAAATATTTTCT[C/T]TCAGTCGGTGGCTTATTTATTCATTTTTCCTTAATTTTTCTTTTGAAGGGAAGTGTTAATTTTTATAAACTCTAATTTATCCTTATAAAAATGAAACCATTTTTATGGTTTGGGCCTTTTTTTTTTTTGGCTCATCTATTAAATTTTTGCCTACCCCAAGTTCATGAACATTTTCT | AAAAATGAATAAATAAGCCACCGACTGAG | AAAAATGAATAAATAAGCCACCGACTGAA | ACCCATAAAATGAAAAACAAAATACTTCTTAGAAAACAAA | CACTTCCCTTCAAAAGAAAAATTAAGGA |  |
| **rs3813948** | AG | A | G | GTCTTCACAGTAATAACTAATGGTGGATCCTAAGGTGAARTTATTTCCTTCAAAATAGCCATGAACTGGATTCCCAGGAGGGTCACAGTCCCCTGAGAGA[A/G]ACRAGTATAGAAGTCATAGTTATGAGGGCCAGRCACGCTCAATGAACAGCACCCCTAATTGCAGCTGTTTATGCCAGCAYCTGACAGCCTCAGAGCAACA | GTCACAGTCCCCTGAGAGAA | GTCACAGTCCCCTGAGAGAG | CTGGCCCTCATAACTATGACTTC | AGCCATGAACTGGATTCCCA |  |
| **rs4253399** | GT | G | T | TGAACACAGAGAGCGGCGGGGCCGGCGGGGAAGGAGGGAGGAGGGGAGAGCGCTGCTTCCCYGTGGGTTCCGGCTTCTGCAGAGCTGTAAGAGT[G/T]GAATGCCACACACAGTCACACTAAGGAATGCTCCAGGATTGGGAAAGAAAATTCAACNTTATAATGAGAACACTGTGAATGCTATTGAATTAACTACTCC | GTGACTGTGTGTGGCATTCC | TGTGACTGTGTGTGGCATTCA | GTGGGTTCCGGCTTCTGC | CCAATCCTGGAGCATTCCTT |  |
| **rs4253417** | CT | C | T | ATTTCTTTGATTATCAAAAGTACACTAGCTAAGGTTGCTGTCCCTCTTTTATTTATTTATTTATTTGAGACAGGGTCTTGCTCTGTCACTCAGATTTGGT[C/T]GCACTGGGTGTGATCTCAGCCCACTGCAGCCTCCACTTCCTAGGCTCAAGCAATCCACCCGTCTCATCCTCCCAAGTTGCTGGGACCACAGGTGTGCACC | TGAGATCACACCCAGTGCG | CTGAGATCACACCCAGTGCA | GAGACAGGGTCTTGCTCTGTCA | AGGCTGCAGTGGGCT |  |
| **rs4524** | AG | A | G | AGCATTCCAGCCCATATTCTGAAGACCCTATAGAGGATCCTCTACAGCCAGATGTCACAGGGATACGTCTACTTTCACTTGGTGCTGGAGAATTCA[A/G]AAGTCAAGAACATGCTAAGCATAAGGGACCCAAGGTAGAAAGAGATCAAGCAGCAAAGCACAGGTTCTCCTGGATGAAATTACTAGCACATAAAGTTGGG | TCACTTGGTGCTGGAGAATTCAA | CACTTGGTGCTGGAGAATTCAG | GAGAACCTGTGCTTTGCTGCT | TGTCACAGGGATACGTCTACTTT |  |
| **rs529565 (v2)** | CT | C | T | CTCCAAACCTGTTTTCCCGTGGGTGAAAGAATGACCCGGGAAGTATTTACCGTTCCTTCCTGAGAACTCAGCGATA[C/T]TGAACACAGTGCTGCCTCACAGTAAACACTGACAAATGGTGAGCATTACTGAGGGCAGGGCCTCGACCTACACCATACCCAGTTTTCTTCATGAATTTTCCACTCCTCTGCCTCCCGCTCCTCCAGG | CCTTCCTGAGAACTCAGCGATAC | CCTTCCTGAGAACTCAGCGATAT | TGTGAGGCAGCACTGTGTTCA | TGAAAGAATGACCCGGGAAGTA |  |
| **rs5918** | CT | C | T | TTAGCTATTGGGAAGTGGTAGGGCCTGCAGGAGGTAGAGAGTCGCCATAGYTCTGATTGCTGGACTTCTCTTTGGGCTCCTGTCTTACRGGCCCTGCCTC[C/T]GGGCTCACCTCGCTRTGACCTGAAGGAGAATCTGCTGAAGGATAACTGTGCCCCAGAATCCATCGAGTTCCCAGTGAGTGAGGCCCGAGTACTAGAGGAC | AGCGAGGTGAGCCCG | AGCGAGGTGAGCCCA | CTGGACTTCTCTTTGGGCTCCT | AGCAGATTCTCCTTCAGGTCA |  |
| **rs5985** | GT | G | T | CGCCTTTGGAGGCAGAAGAGCAGTTCCACCCAATAACTCTAATGCAGCGGAAGATGACCTGCCCACAGTGGAGCTTCAGGGC[G/T]TGGTGCCCCGGGGCGTCAACCTGCAAGGTATGAGCATACCCCCCTTCCCCACCACTCTGGGTCCA | AGTGGAGCTTCAGGGCG | CAGTGGAGCTTCAGGGCT | CTTGCAGGTTGACGCCCC | TGCAGCGGAAGATGACCT |  |
| **rs6025** | AG | A | G | TTAACAAGACCATACTACAGTGACGTGGACATCATGAGAGACATCGCCTCTGGGCTAATAGGACTACTTCTAATCTGTAAGAGCAGATCCCTGGACAGGC[A/G]AGGAATACAGGTATTTTGTCCTTGAAGTAACCTTTCAGAAATTCTGAGAATTTCTTCTGGCTAGAACATGTTAGGTCTCCTGGCTAAATAATGGGGCATT | ACTTCAAGGACAAAATACCTGTATTCCTT | CTTCAAGGACAAAATACCTGTATTCCTC | CTGTAAGAGCAGATCCCTGGACA | TGTTCTAGCCAGAAGAAATTCTCAGA |  |
| **rs6536024** | CT | C | T | ATACACATTATATATACATATACATACATATGAAACCTTTGCTGTTAGATATGTCAGATAATACAGAATGAGTGGTAATCATTTAACACATTTCATGTTC[C/T]GTGGTGCCAGAAATGGGTAGGCTGTCTAGTAAATAGATTTTGTTAATTAGTACATTCTGTGATATAATCTAGTTGTGCTGAATTTCCTGAGATGCTTGGA | AGTGGTAATCATTTAACACATTTCATGTTCC | AGTGGTAATCATTTAACACATTTCATGTTCT | GCCTACCCATTTCTGGCACC | GCTGTTAGATATGTCAGATAATACAGAATGA |  |
| **rs710446** | AG | A | G | TGGGGAGGTCGTGTCTGGAAAATCTGATATTGGGTTAAATGAAAGGCCATTTGGGTCTATCTGGATATCAGGGATCCAATCGTCATCACTCTGT[A/G]TGGGAGCTGGTGATATAGGAGGCATCATAGTTGCAATGAGATCAGAGTCCTGAAAGTCAGAAAAGGTAACTGTTACACCTGGCTTGGCTAGGGAAGGGAT | GGATCCAATCGTCATCACTCTGTA | GGATCCAATCGTCATCACTCTGTG | CCTCCTATATCACCAGCTCCCA | CCATTTGGGTCTATCTGGATATCAG |  |
| **rs8176592** | CT | C | T | ATTAAATACCCCTTTATTCAGATTACTGTTTTACATACACATGCAACAACATTAATCTATAAACAATATACAATACGAAAACCTGAAATCCACTATCACA[C/T]ATGGCTTACCATGTTTTCCTGATTGTTTTTAGAATTTCACGGTCCCTCATGGTGTCTCACTCCAGCAGACAGAGGATTGTGTCGTGCCAATGAGAACAGA | CGAAAACCTGAAATCCACTATCACAC | CGAAAACCTGAAATCCACTATCACAT | ACACCATGAGGGACCGTGAA | ACGAAAACCTGAAATCCACTATCAC |  |
| **rs8176719** | [-/G] | - | G | AGAGGCRCATGTGGGTGGCACCCTGCCAGCTCCATGTGRCCGCACGCCTCTCTCCATGTGCAGTAGGAAGGATGTCCTCGTGGT[/G]ACCCCTTGGCTGGCTCCCATTGTCTGGGAGGGCACATTCAACATCGACATCCTCAACGAGCAG | GAGCCAGCCAAGGGGTA | AGCCAGCCAAGGGGTC | CGCACGCCTCTCTCCATGT | GTGCCCTCCCAGACAATG |  |
| **Legend:**  **ASP1** = SNP allele detected with allele-specific primer 1; **ASP2** = SNP allele detected with allele-specific primer 2; **SNP_SEQ** = sequence of the amplified fragment containing the SNP; **ASP1_SEQ** = sequence of allele-specific primer 1; **ASP2_SEQ** = sequence of allele-specific primer 2; **LSP_SEQ** = sequence of locus-specific reverse primer; **STA_SEQ** = sequence of forward primer for specific target amplification; | | | | | | | | |  |
