# Supplementary material for: Genetic association study of fatal pulmonary embolism
Source: Int J Legal Med. 2020 Oct 30;135(1):143–51. doi: 10.1007/s00414-020-02441-7 (PMC7782449; doi:10.1007/s00414-020-02441-7)
Supplement: Supplementary file 2 — (DOCX 18 kb) [file 414_2020_2441_MOESM2_ESM.docx]

Table S2: Comparison between MAFs cited in literature and the control group of the present study

| **Gene** | **refSNP acession no.** | **Effect allele** | **Expected MAF** | **MAF in control group** |
| --- | --- | --- | --- | --- |
| *MTHFR* | **rs1801133** | T | 0.35 | 0.35 |
| *MTHFR* | **rs1801131** | C | 0.32 | 0.31 |
| *F5* | **rs6025** | A | 0.03 | 0.03 |
| *F2* | **rs1799963** | A | 0.01 | 0.02 |
| *FGB* | **rs1800790** | A | 0.15 | 0.26 |
| *F13A1* | **rs5985** | A | 0.24 | 0.3 |
| *ITGB3* | **rs5918** | C | 0.16 | 0.15 |
| *TFPI* | **rs8176592** | C | 0.33 | 0.33 |
| *TFPI* | **rs10931292** | C | 0.14 | 0.13 |
| *F5* | **rs4524** | G | 0.28 | 0.29 |
| *FGG* | **rs2066865** | T | 0.27 | 0.26 |
| *FGG* | **rs6536024** | C | 0.47 | 0.46 |
| *ABO* | **rs8176719** | deletion | 0.39 | 0.43 |
| *ABO* | **rs2519093(v2)** | A | 0.17 | 0.24 |
| *ABO* | **rs529565(v2)** | C | 0.39 | 0.42 |
| *F11* | **rs4253399** | G | 0.31 | 0.4 |
| *F11* | **rs4253417** | C | 0.33 | 0.42 |
| *F11* | **Rs2036914** | C | 0.41 | 0.48 |
| *SLC44A2* | **rs2288904** | T | 0.2 | 0.19 |
| *HIVEP1* | **rs169713** | C | 0.31 | 0.2 |
| *C4BPB* | **rs3813948** | G | 0.09 | 0.07 |
| *TC2N* | **rs1884841** | T | 0.46 | 0.43 |
